# A Systematic Review of Inflammatory Markers in Polycystic Ovary Syndrome (PCOS) and Meta-Analysis of Interleukin-6 (IL-6) in Case-Control Studies

**DOI:** 10.7759/cureus.82350

**Published:** 2025-04-16

**Authors:** Bhavit Bansal, Avelyn Thazhuthadath Kishore, Sasikala Kathiresan, Arzina Farook Ghachi, Swetapadma Pradhan, Sheuli Paul, Khyati Chaturvedi, Mukul Singh, Sauvit Patil, Lalitha Soumya Johnson, Akshay V P, Delna NS, Ajita Pillai

**Affiliations:** 1 Research, Central Council for Research in Yoga and Naturopathy, Delhi, India; 2 Plastic and Reconstructive Surgery, Jubilee Mission Medical College and Research Institute, Thrissur, IND; 3 Obstetrics and Gynaecology, All India Institute of Medical Sciences, Madurai, IND; 4 Internal Medicine, Shroff Hospital, Vadodara, IND; 5 Medical College, European University Faculty of Medicine, Tbilisi, GEO; 6 Paediatrics, Dr. D. Y. Patil Medical College, Hospital and Research Centre, Dr. D. Y. Patil Vidyapeeth (Deemed to be University), Pimpri, IND; 7 Naturopathy and Yogic Science, Sant Hirdaram Medical College of Naturopathy and Yogic Sciences for Women, Bhopal, IND; 8 Surgery, All India Institute of Medical Sciences, Gorakhpur, IND; 9 Molecular Neuroscience, Bristol Medical School, Faculty of Health and Life Sciences, University of Bristol, Bristol, GBR; 10 Biotechnology and Microbiology, Dr. Janaki Ammal Campus, Kannur, IND; 11 Biomedical Research and Molecular Biology, Mansarovar Global University, Bhopal, IND; 12 Biomedical Sciences, BioDeskINDIA Labs, Bhopal, IND; 13 Allied Health Sciences, Al-Azhar Medical College and Super Specialty Hospital, Thodupuzha, IND

**Keywords:** female reproductive health, inflammatory bio markers, polycystic ovary syndrome (pcos), systematic review and meta analysis, women’s health

## Abstract

Chronic low-grade inflammation plays a crucial role in the pathophysiology of polycystic ovary syndrome (PCOS). This systematic review aims to synthesize the recent evidence on key inflammatory markers in PCOS and the role of IL-6.

A comprehensive literature search was conducted using the PubMed, Web of Science, Cochrane Library, and Google Scholar databases. Studies published between 2014 and 2024 were screened based on predefined eligibility criteria. Both observational and interventional studies that reported levels of inflammatory markers in patients with polycystic ovary syndrome (PCOS) were included. Data were systematically extracted, and the quality of the studies was assessed using the Newcastle-Ottawa Scale (NOS) for observational studies, the Cochrane Risk of Bias tool for randomized controlled trials (RCTs), and the ROBINS-1 tool for non-randomized controlled trials. A statistical synthesis of IL-6 levels was performed for the meta-analysis using a random-effects model in R.

A total of 44 studies met the inclusion criteria for qualitative analysis and identified 94 biomarkers. The most commonly used biomarkers across the majority of studies, listed in descending order, are as follows: high-sensitivity C-reactive protein (hs-CRP), interleukin-6 (IL-6), tumor necrosis factor-alpha (TNF-α), CRP, adiponectin, IL-18, vascular endothelial growth factor (VEGF), IL-8, IL-1β, sex hormone binding globulin (SHBG), leptin, and vascular cell adhesion protein 1 (VCAM-1). Additionally, four case-control studies conducted in four countries (Taiwan, Russia, Spain, and Turkey) were included in the quantitative analysis, which involved 689 participants (PCOS group: n = 365; Control group: n = 324). The pooled mean difference (MD), calculated using the random-effects model, was 0.72 (0.47; 0.98) (p < 0.0001), indicating a significant increase in IL-6 levels among PCOS patients compared to the control group. The funnel plot exhibited slight asymmetry, suggesting publication bias, where smaller studies with negative or neutral results may be absent. The adjusted effect size after trim and fill analysis remained significant, indicating that publication bias is unlikely to affect the conclusions substantially.

Chronic low-grade inflammation plays a crucial role in PCOS, and IL-6 levels in women with PCOS were elevated. Potential markers that can be investigated to assess inflammatory status in PCOS include hs-CRP, TNFα, CRP, adiponectin, IL-18, VEGF, IL-8, iIL-1β, SHBG, leptin, and VCAM-1.

## Introduction and background

Polycystic ovary syndrome (PCOS) is a complex and heterogeneous endocrine disorder affecting 5-15% of reproductive-aged women worldwide. It is characterized by a spectrum of metabolic, reproductive, and psychological abnormalities, including hyperandrogenism, ovulatory dysfunction, polycystic ovarian morphology, insulin resistance, obesity, and increased cardiovascular risk [1,2].

Chronic low-grade inflammation has been increasingly recognized as a central component in PCOS-related metabolic dysfunction. Current studies have demonstrated that there is a heightened systemic inflammatory state in PCOS, and this persistent inflammatory burden is believed to contribute to insulin resistance, dyslipidemia, endothelial dysfunction, and cardiovascular complications, all of which are very common in PCOS [3]. In addition to this, inflammation is found to play a role in ovarian dysfunction by disrupting follicular development, steroidogenesis, and oocyte quality, thereby exacerbating reproductive abnormalities in affected women [4].

Exploring this relationship between inflammatory markers in PCOS is an active area of research, and there are several studies conducted on this objective from the past decade [5]. Even though studies are being conducted continuously in this domain, there is a lack of consensus on which inflammatory markers are most relevant in understanding disease mechanisms and guiding therapeutic interventions. 

Our current systematic review aims to fill these gaps by critically analyzing and summarizing the existing evidence on inflammatory markers in PCOS. It will provide a comprehensive evaluation of their measurement frequency, clinical utility, and potential as diagnostic and prognostic tools.

Another systematic review of inflammatory markers in PCOS was published in 2010; it predated the implementation of the Preferred Reporting Items for Systematic Reviews and Meta-Analyses (PRISMA) guidelines. Since 2010, research has expanded, and new findings highlight the role of inflammation in PCOS with a stronger focus on inflammatory markers and their links to insulin resistance, obesity, and cardiovascular risk [1,3].

This review aims to update the literature by synthesizing evidence from studies published between 2014 and 2024. By following PRISMA guidelines, the study will provide a rigorous, standardized assessment of the evidence linking PCOS with key inflammatory markers. This updated synthesis will inform clinicians and researchers about the current understanding of inflammation in PCOS and guide future research and treatment approaches.

## Review

Materials and methods

This systematic review followed the Preferred Reporting Items for Systematic Reviews and Meta-Analyses (PRISMA) guidelines. The complete protocol and supplementary files related to the study are available in the Open Science Framework (OSF) registries (DOI: 10.17605/OSF.IO/W7C3R). This systematic review was registered in the International Prospective Register of Systematic Reviews (PROSPERO) (CRD42024609883).

Inclusion and Exclusion Criteria

The inclusion and exclusion criteria for this systematic review were developed using the PICO (Population, Intervention/Exposure, Comparison, Outcome) framework. Accordingly, a literature search was conducted across various scholarly sources, including but not limited to PubMed, Web of Science, the Cochrane Library, and Google Scholar. This review focused on observational and interventional studies involving women diagnosed with polycystic ovary syndrome (PCOS) based on standardized criteria (e.g., Rotterdam, NIH, Androgen Excess Society) that reported inflammatory markers. The literature search covered studies published from January 1, 2014, to December 31, 2024. Only publications in English were included.

Study Selection

The articles were reviewed independently by two authors, and any disagreements were resolved through consensus with a third author. The reasons for excluding studies were documented. Data extraction was performed independently by three authors using data extraction forms. The extracted data included the authors, year of publication, study design, mean age of participants, diagnostic criteria used, and primary outcomes (inflammatory markers).

Quality Assessment

Risk of bias and quality assessment were conducted using the Cochrane Risk of Bias Tool (Cochrane Collaboration, London, UK) for randomized controlled trials, the ROBINS-1 tool (Cochrane Collaboration) for non-randomized controlled trials, and the Newcastle-Ottawa Scale (Universities of Newcastle, Australia and Ottawa, Canada) for observational studies (Appendices).

Statistical Analysis

All statistical analyses were conducted using R version 4.2.2 (RStudio Team (2020). RStudio: Integrated Development for R. RStudio, PBC, Boston, MA URL http://www.rstudio.com/). Data arrangement and codes were adopted from "Meta-analysis with R" by Schwarzer et al. [6]. The primary outcome measure was the mean difference (MD) between IL-6 levels in the PCOS and control groups.

For studies that reported IL-6 concentrations as medians with interquartile ranges (IQR) rather than means with standard deviations (SD) or standard errors (SE), we estimated the mean and SD using the "Meta-Analysis Accelerator" tool. A random-effects model (DerSimonian and Laird model) was chosen due to the expected variability across studies in terms of population characteristics, sample sizes, and measurement methods. In between the studies, heterogeneity was evaluated using Cochran’s Q test with a significant Q statistic (p < 0.05), suggesting substantial heterogeneity.

I² values were interpreted as I² < 25%, indicating low heterogeneity; I² = 25-50% indicates moderate heterogeneity; and I² > 75%, indicating high heterogeneity. Funnel plots and Egger’s regression test were conducted to detect publication bias statistically. A trim-and-fill analysis was performed to adjust for potential missing studies. To ensure the robustness of the results, a leave-one-out sensitivity analysis was performed, where each study was removed one by one to examine its impact on the overall effect size.

Results

**Figure 1:**
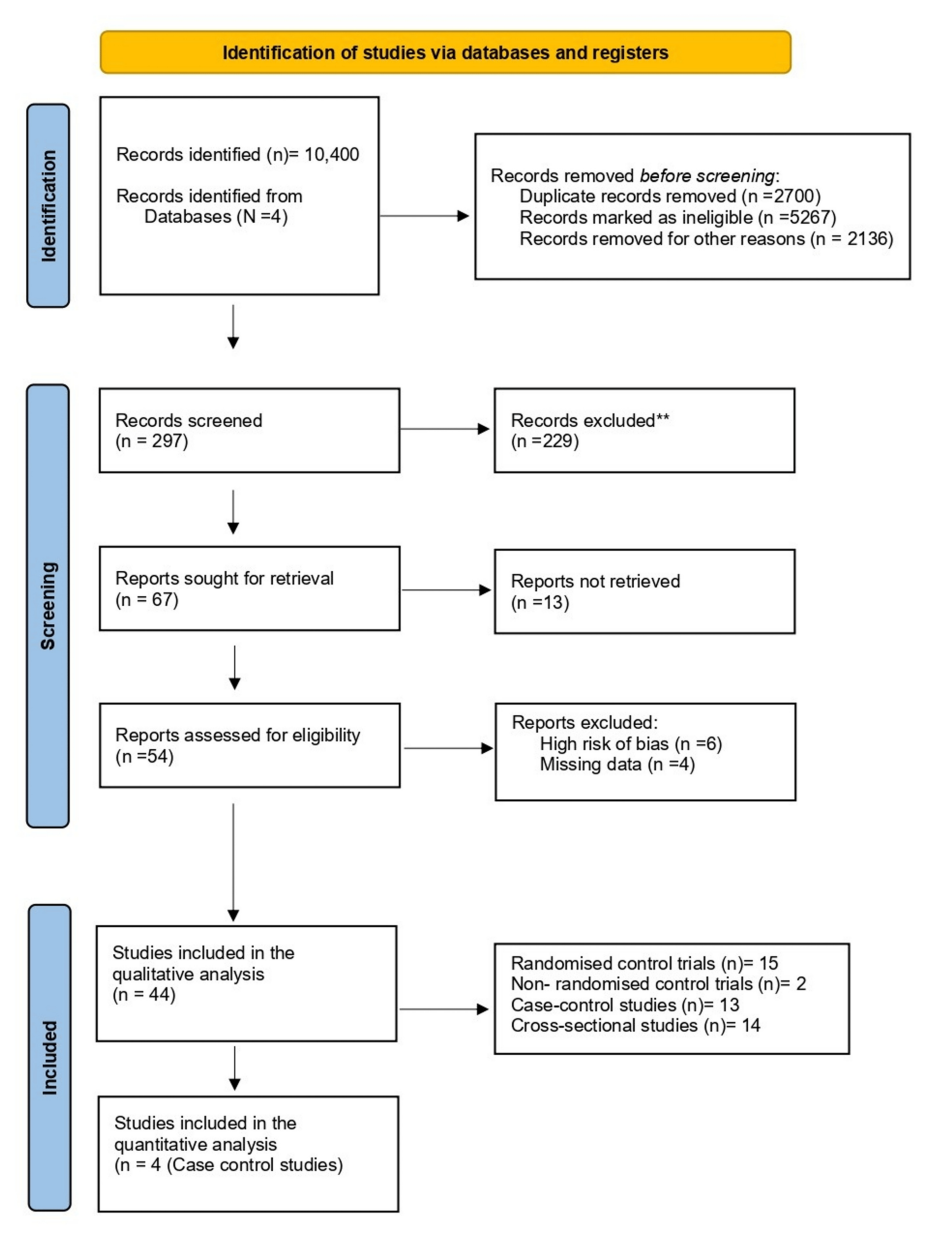
Preferred Reporting Items for Systematic Reviews & Meta-analysis (PRISMA) flow diagram of the study selection process

The systematic literature search was conducted according to the registered protocol. The search identified 10,400 records from four databases: PubMed, Web of Science, Cochrane Library, and Google Scholar. From these records, 297 were screened, and 54 reports were selected for quality assessment and risk-of-bias evaluation. Ultimately, data from 44 studies were included in the qualitative analysis, which consisted of 15 randomized control trials, 2 non-randomized control trials, 13 case-control studies, and 14 cross-sectional studies. From these, four observational studies that reported IL-6 in MD between the PCOS group and control group were selected for quantitative analysis (Tables 1-4). Figure 1 summarizes the PRISMA flow diagram of the study selection process.

**Table 1:** Data extraction table for randomized control trials [7-21] Abbreviations: PCOS: Polycystic Ovary Syndrome; PPAR-γ: Peroxisome Proliferator-Activated Receptor Gamma; IL: Interleukin; MCP-1: Monocyte Chemoattractant Protein-1; MCP: Monocyte Chemoattractant Protein; hs-CRP: High-Sensitivity C-Reactive Protein; CRP: C-Reactive Protein; NF-κB: Nuclear Factor Kappa-Light-Chain-Enhancer of Activated B Cells; VEGF: Vascular Endothelial Growth Factor; PIGF: Placental Growth Factor; MDA: Malondialdehyde; CAT: Catalase; SOD: Superoxide Dismutase; SHBG: Sex Hormone-Binding Globulin; MIP: Macrophage Inflammatory Protein; CXCL: C-X-C Motif Chemokine Ligand; GRP78: Glucose-Regulated Protein 78; sXBP1: Spliced X-Box Binding Protein 1; ATF6: Activating Transcription Factor 6; VCAM-1: Vascular Cell Adhesion Molecule-1; ICAM-1: Intercellular Adhesion Molecule-1; E-Selectin: Endothelial Selectin; RBP-4: Retinol-Binding Protein 4; DPP-IV: Dipeptidyl Peptidase IV

AUTHOR, YEAR	COUNTRY	SAMPLE SIZE	INFLAMMATORY MARKERS
Jamilian et al., 2017 [7]	Iran	Intervention= 20, Placebo= 20	PPAR-γ, IL-8, TNF-α, TGF-β
Sathyapalan et al., 2017 [8]	UK	20 patients with PCOS	VEGF, TNFα, IL-1β, IL-1ra, IL-2, IL-6, IL-8, IL-10, MCP-1
Abbasi et al., 2023 [9]	Iran	57 PCOS patients	hs-CRP
Brenjian et al., 2019 [10]	Iran	40 patients with PCOS were divided into two groups	IL-6, IL-1β, IL-18, TNF-α, NF-Κb, CRP
Jabarpour et al., 2024 [11]	Iran	56 PCOS patients	TNF-α, IL-18, IL-6, CRP, Active Caspase-3, Caspase-8
Elbandrawy et al., 2022 [12]	Egypt and Saudi Arabia	40 patients with PCOS were divided into two groups	IL-6, TNF-α, CRP
Fereidouni et al., 2024 [13]	Iran	44 infertile Polycystic Ovary Syndrome (PCOS) patients.	IL-6, IL-1β, MDA, CAT, SOD
Rahimi et al., 2022 [14]	Iran	50 women with PCOS were divided into two groups (n = 25 each)	IL-6, TNF-α
Verma et al., 2024 [15]	India	PCOS group: 30, Control group: 30	hs-CRP
Asemi et al., 2015 [16]	Iran	PCOS group: 24, Control group: 24	Serum insulin, HOMA-IR, hs-CRP
Heidar et al., 2020 [17]	Iran	PCOS group: 18, Control group: 18	IL-1, TNF-α, VEGF, IL-8, TGF-β
Nasri et al., 2018 [18]	Iran	60 PCOS subjects	SHBG, hs-CRP
Esmaeilinezhad et al.,2020 [19]	Iran and Canada	86 participants	TAC, MDA, hs-CRP
Zangeneh et al., 2017 [20]	Iran	85 PCOS, 86 healthy women	IL-1α, IL-1β, IL-1, TNFα
Alissa et al., 2021 [21]	Saudi Arabia	Men with and without PCOS	TNF-α, CRP, IL-6

**Table 2:** Data extraction table for non-randomized control trials [22,23] Abbreviations: PCOS: Polycystic Ovary Syndrome; hs-CRP: High-Sensitivity C-Reactive Protein; TNF-α: Tumor Necrosis Factor-Alpha

AUTHOR, YEAR	COUNTRY	SAMPLE SIZE	INFLAMMATORY MARKERS
Covington et al., 2014 [22]	USA	8 PCOS women and 8 obese control females	hs-CRP, TNF-α, total and high molecular weight adiponectin
Szczuko et al., 2017 [23]	Poland	36 women (control group+ PCO high testosterone+ PCO testosterone normal)	6 (R), 15(R)-lipoxin A4 5(S), 6(R)-lipoxin A4, 16(R)-HETE, 16(S)-HETE, 13(S)-HODE, 9(S)-HODE, 15(S)-HETE, 12(S)-HETE, 5(S)-oxoETE, 5(S)-HETE

**Table 3:** Data extraction table of case-control studies [24-36] Abbreviations: PCOS: Polycystic Ovary Syndrome; CRP: C-Reactive Protein; SHBG: Sex Hormone-Binding Globulin; hs-CRP: High-Sensitivity C-Reactive Protein; IL: Interleukin; MIP-1α: Macrophage Inflammatory Protein-1 Alpha; TNF-α: Tumor Necrosis Factor-Alpha; FKN: Fractalkine; MCP: Monocyte Chemoattractant Protein; CXCL: C-X-C Motif Chemokine Ligand; GRP78: Glucose-Regulated Protein 78; sXBP1: Spliced X-Box Binding Protein 1; ATF6: Activating Transcription Factor 6; VCAM-1: Vascular Cell Adhesion Molecule-1; ICAM-1: Intercellular Adhesion Molecule-1; E-Selectin: Endothelial Selectin; G-CSF: Granulocyte Colony-Stimulating Factor

AUTHOR, YEAR	COUNTRY	SAMPLE SIZE	INFLAMMATORY MARKERS
Rudnicka et al., 2020 [24]	Poland	PCOS: 200, Control: 105	CRP, SHBG
Shen et al., 2015 [25]	Taiwan	165 women with PCOS and 165 women without PCOS	hs-CRP, IL-6, Adiponectin, Leptin, Ghrelin, Resistin, SHBG
Keskin et al., 2014 [26]	Turkey	120 women of reproductive age without PCOS with PCOS (60/60), PCOS group (obese = 32, lean = 30)	hs-CRP
Vasyukova et al., 2023 [27]	Russia	44 women with PCOS (19 with BMI < 25, 25 with BMI ≥ 25), 45 controls (22 with BMI < 25, 23 with BMI ≥ 25)	IL-1ra, IL-2, IL-6, IL-17E, IL-17A, IL-18, MIP-1α, IL-1α, IL-4, IL-9, IL-12, IL-13, IL-15, TNF-α, SCD40L, FKN, MCP-3, MIP-1β, IL-22
Pedroso et al., 2014 [28]	Brazil	150 PCOS, 124 controls	CRP
Demir et al., 2019 [29]	Turkey	80 PCOS, 80 Controls	hs-CRP, FKN
Wang et al., 2017 [30]	China	44 Clomiphene citrate-resistant, 55 Clomiphene citrate-sensitive PCOS patients	hs-CRP, CXCL-16, Angiopoietin-2
Bañuls et al., 2017 [31]	Spain	148 PCOS (116 without metabolic syndrome, 32 with metabolic syndrome), 112 controls (87 without metabolic syndrome, 25 with metabolic syndrome)	GRP78, sXBP1, ATF6, VCAM-1, ICAM-1, E-Selectin, TNF-α, IL-6
Apaydın et al., 2021 [32]	Türkiye	52 PCOS patients, 59 controls	Serum, endocan
Niu et al., 2017 [33]	China.	60 PCOS patients (PCOS non-metabolic syndrome and PCOS metabolic syndrome) and 30 controls	TNF-α, G-CSF
Akan et al., 2022 [34]	Turkey	75 PCOS subjects 75 age and BMI-matched controls	Dermcidin, hs-CRP
Ganie et al., 2016 [35]	India (Kashmir)	220 PCOS cases 220 age-matched non-PCOS healthy controls	ICAM-1
Eser et al., 2017 [36]	Turkey	76 PCOS and 27 controls	IL-1A, IL-6

**Table 4:** Data extraction table for cross-sectional studies [37-50] Abbreviations: PCOS: Polycystic Ovary Syndrome; PPAR-γ: Peroxisome Proliferator-Activated Receptor Gamma; IL: Interleukin; MCP: Monocyte Chemoattractant Protein; hs-CRP: High-Sensitivity C-Reactive Protein; CRP: C-Reactive Protein; NF-κB: Nuclear Factor Kappa-Light-Chain-Enhancer of Activated B Cells; VEGF: Vascular Endothelial Growth Factor; PIGF: Placental Growth Factor; MDA: Malondialdehyde; CAT: Catalase; SOD: Superoxide Dismutase; SHBG: Sex Hormone-Binding Globulin; MIP: Macrophage Inflammatory Protein; CXCL: C-X-C Motif Chemokine Ligand; GRP78: Glucose-Regulated Protein 78; sXBP1: Spliced X-Box Binding Protein 1; ATF6: Activating Transcription Factor 6; VCAM-1: Vascular Cell Adhesion Molecule-1; ICAM-1: Intercellular Adhesion Molecule-1; E-Selectin: Endothelial Selectin; RBP-4: Retinol-Binding Protein 4; DPP-IV: Dipeptidyl Peptidase IV; MMP-1: Matrix Metalloproteinase; S100A8: S100 Calcium-Binding Protein A8; sICAM-1: Soluble Intercellular Adhesion Molecule-1

AUTHOR, YEAR	COUNTRY	SAMPLE SIZE	INFLAMMATORY MARKERS
Goswami et al., 2021 [37]	India	30 women with PCOS, 30 healthy controls	hs-CRP, IL-6, TNFα, PMP
Daan et al., 2016 [38]	Netherland	34 hyperandrogenic PCOS, 34 normoandrogenic PCOS women, 32 non-PCOS women, 14 PCOS offspring, 30 pediatric reference	Leptin, Adiponectin, RBP-4, DPP-IV, MMP-9, S100A8, IL-6, TNF-α, IL-1b, IL-18, IL-13, IL-17, CCL2/MCP-1, PIGF, VEGF, sICAM-1, sVCAM-1, sVEGF-R1, MMP-9, S100A8, Cathepsin S
Kałużna et al., 2020 [39]	Poland	395 women of reproductive age (18–40 age) with (n = 270) and without (n = 125) PCOS	LMR, MHR, hs-CRP
Tola et al., 2017 [40]	Turkey	A total of 67, 34 as PCOS and 33 BMI-matched controls	NEO, CRP
Hatziagelaki et al., 2020 [41]	Greece	63 women with PCOS	CRP, CXCL11, CCL4, MCP-4/CCL13, CXCL5, CXCL6, VEGF, LAP-TGFβ1, TNFSF14, MMP-1, CXCL1, MCP-2/CCL8, CDCP1, CST5, CSF-1
Ramamoorthy et al., 2019 [42]	India	Total = 90, 30 in each group, total 3 groups (Group 1: Healthy volunteers, Group 2: Newly diagnosed PCOS, Group 3: Already on treatment for PCOS)	hs-CRP, IL-6, IL-18
Giallauria et al., 2019 [43]	Italy	243 PCOS patients	CRP
Shorakae et al., 2018 [44]	Australia	49 PCOS, 23 controls	hs-CRP, HMW-adiponectin
Atnil et al., 2022 [45]	Indonesia	45 lean PCOS women with normal BMI (18.5–22.9 kg/m²)	MHR (Monocyte-HDL ratio)
Asfuroğlu et al., 2021 [46]	Turkey	47 PCOS patients, 43 control patients with unexplained infertility	DHEA-S
Bicer et al., 2017 [47]	Turkey	80 women with PCOS, 80 controls	hs-CRP, Endocan
Amjadi et al., 2015 [48]	Iran	10 PCOS patients, 15 healthy controls	Apolipoprotein A1
Temur et al., 2017 [49]	Turkey	80 women	hs-CRP
Koleva et al., 2016 [50]	Bulgaria	76 PCOS women	Leptin, Adiponectin, sICAM-1, sVCAM-1

In total, 94 markers were identified, and those most frequently used in studies are listed and briefly discussed. The most commonly used biomarkers in the majority of studies, in descending order, are as follows: hsCRP> IL-6> TNFα> CRP> Adiponectin> IL-18> VEGF> IL-8> IL-1β> SHBG> Leptin> VCAM-1. These identified markers can be classified into nine categories (Table 5).

**Table 5:** Classification of inflammatory markers identified in the review Abbreviations: PCOS: Polycystic Ovary Syndrome; PPAR-γ: Peroxisome Proliferator-Activated Receptor Gamma; IL: Interleukin; MCP: Monocyte Chemoattractant Protein; hs-CRP: High-Sensitivity C-Reactive Protein; CRP: C-Reactive Protein; NF-κB: Nuclear Factor Kappa-Light-Chain-Enhancer of Activated B Cells; VEGF: Vascular Endothelial Growth Factor; PIGF: Placental Growth Factor; MDA: Malondialdehyde; CAT: Catalase; SOD: Superoxide Dismutase; SHBG: Sex Hormone-Binding Globulin; MIP: Macrophage Inflammatory Protein; CXCL: C-X-C Motif Chemokine Ligand; GRP78: Glucose-Regulated Protein 78; sXBP1: Spliced X-Box Binding Protein 1; ATF6: Activating Transcription Factor 6; VCAM-1: Vascular Cell Adhesion Molecule-1; ICAM-1: Intercellular Adhesion Molecule-1; E-Selectin: Endothelial Selectin; RBP-4: Retinol-Binding Protein 4; DPP-IV: Dipeptidyl Peptidase IV; MMP-1: Matrix Metalloproteinase; S100A8: S100 Calcium-Binding Protein A8; sICAM-1: Soluble Intercellular Adhesion Molecule-1

Categories	Markers
Cytokines & Interleukins	IL-1, IL-1α, IL-1β, IL-1b, IL-1ra, IL-2, IL-4, IL-6, IL-8, IL-9, IL-10, IL-12, IL-13, IL-15, IL-17, IL-17 A, IL-17 E, IL-18, IL-22
Acute Phase Proteins & Inflammatory Biomarkers	hs-CRP, CRP, NF-κB, TNF-α, TNFα, SCD40L, TNFSF14
Chemokines	MCP-1, MCP-3, MCP-4, MIP-1α, MIP-1β, Fractalkine (FKN), CXCL-1, CXCL-5, CXCL-6, CXCL-11, CXCL-16, CCL-2/MCP-1, CCL-4, CCL-13
Growth Factors	VEGF, PIGF, VEGF-A, sVEGF-R1, Angiopoietin-2
Lipid Mediators	16(S)-HETE, 5(S)-HETE, 5(S)-oxoETE, 12(S)-HETE, 13(S)-HODE, 15(S)-HETE, 9(S)-HODE, 16(R)-HETE, 6(R), 15(R)-lipoxin A4, 5(S), 6(R)-lipoxin A4, 16(R)/16(S)-HETE
Oxidative Stress & Metabolic Markers	MDA, CAT, SOD, Serum insulin, HOMA-IR, ROS
Apoptotic Markers	Active Caspase-3, Active Caspase-8
Metabolic & Adipokines	Leptin, Adiponectin, Ghrelin, Resistin, HMW-adiponectin, SHBG, RBP-4, DPP-IV, Apolipoprotein A1
Enzymes & Proteins	PPAR-γ, MMP-1, MMP-9, S100A8, Cathepsin S, GRP78, sXBP1, ATF6, Dermcidin, NEO

Quantitative Analysis

**Figure 2:**
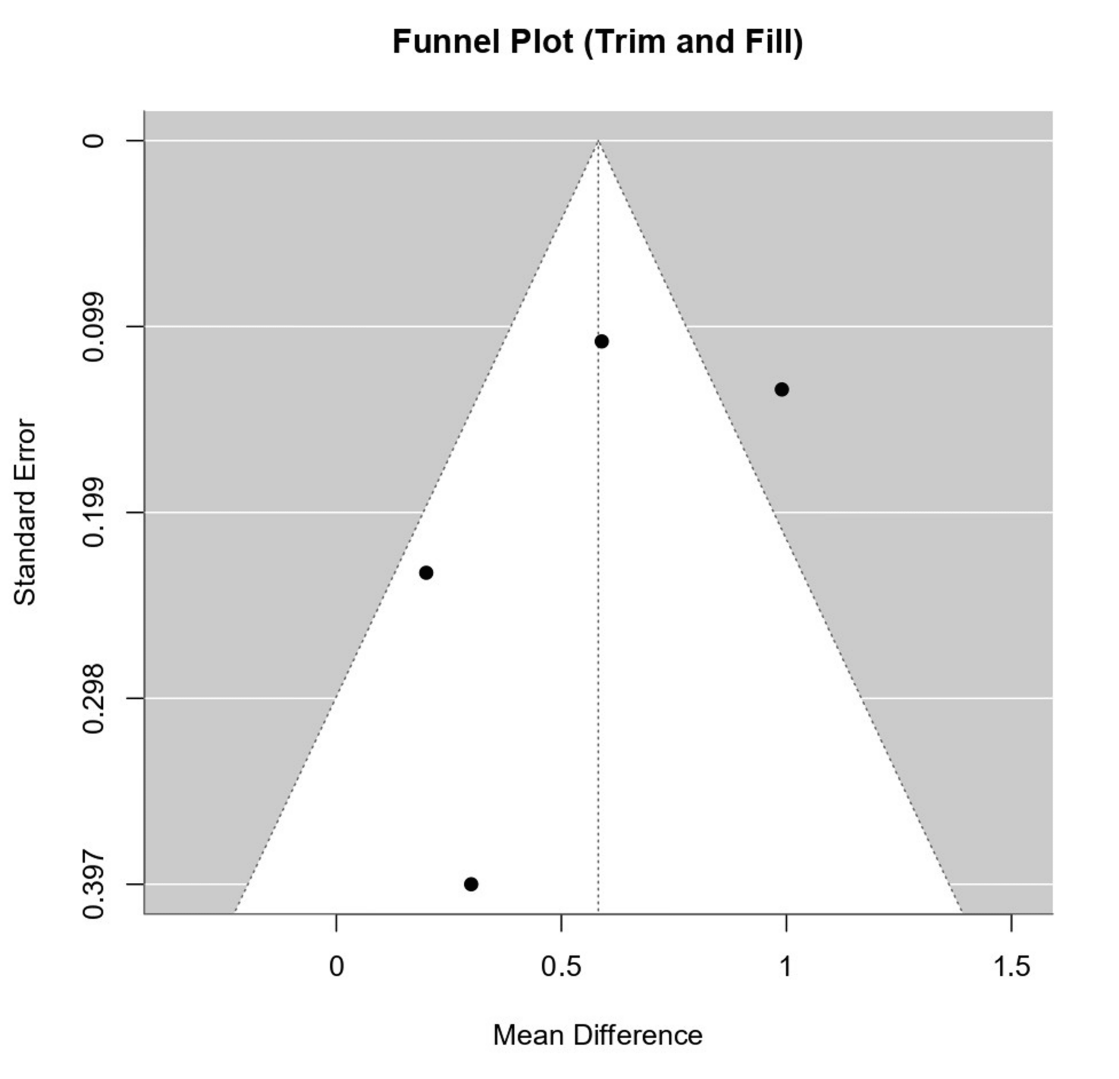
Funnel plot with trim-and-fill analysis assessing publication bias among four studies reporting IL-6 levels in women with PCOS versus controls (mean difference as the effect size). The central vertical line represents the pooled effect size estimate. IL-6: Interleukin-6; PCOS: Polycystic Ovary Syndrome

**Figure 3:**
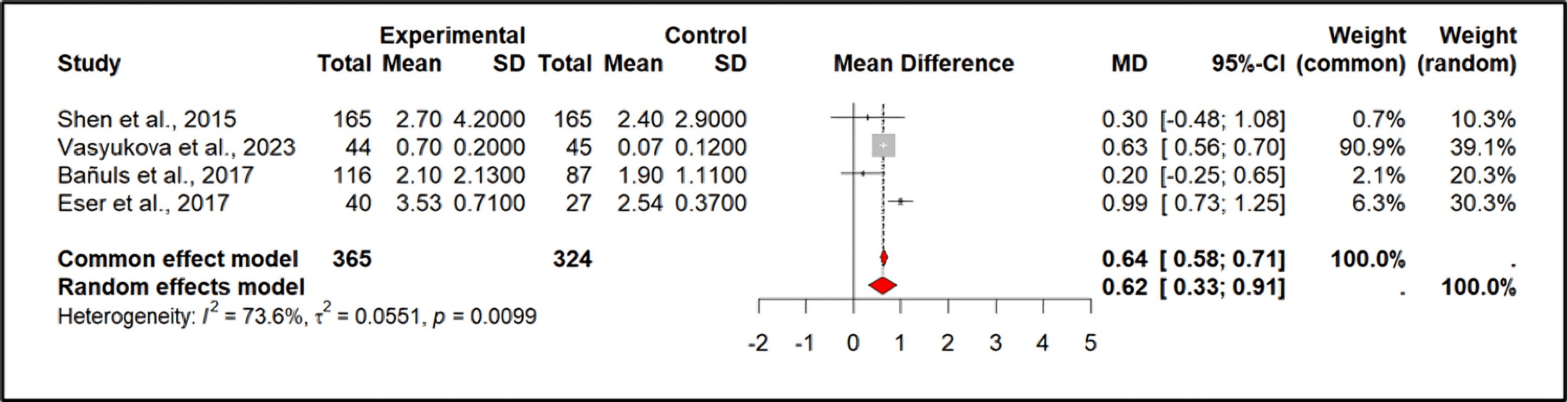
The forest plot summarizes the results of a meta-analysis comparing IL-6 levels in PCOS patients vs. healthy controls across four studies Source: [25,27,31,36] IL-6: Interleukin-6; PCOS: Polycystic Ovary Syndrome

Four observational studies were included in the quantitative analysis comprising 689 participants (PCOS (n) = 365, Control (n) = 324). These studies were conducted across five countries (Taiwan, Russia, Spain, and Turkey). The pooled mean difference (MD) using the random-effects model was 0.72 (0.47; 0.98) (p < 0.0001), indicating a significant increase in IL-6 levels in PCOS patients compared to controls. Individual study estimates varied, with Eser et al. [36] showing the highest MD (0.99 (0.73; 1.25)) while Bañuls et al. [31] had the lowest (0.20 (-0.25; 0.65)) (Figures 2, 3).

**Figure 4:**
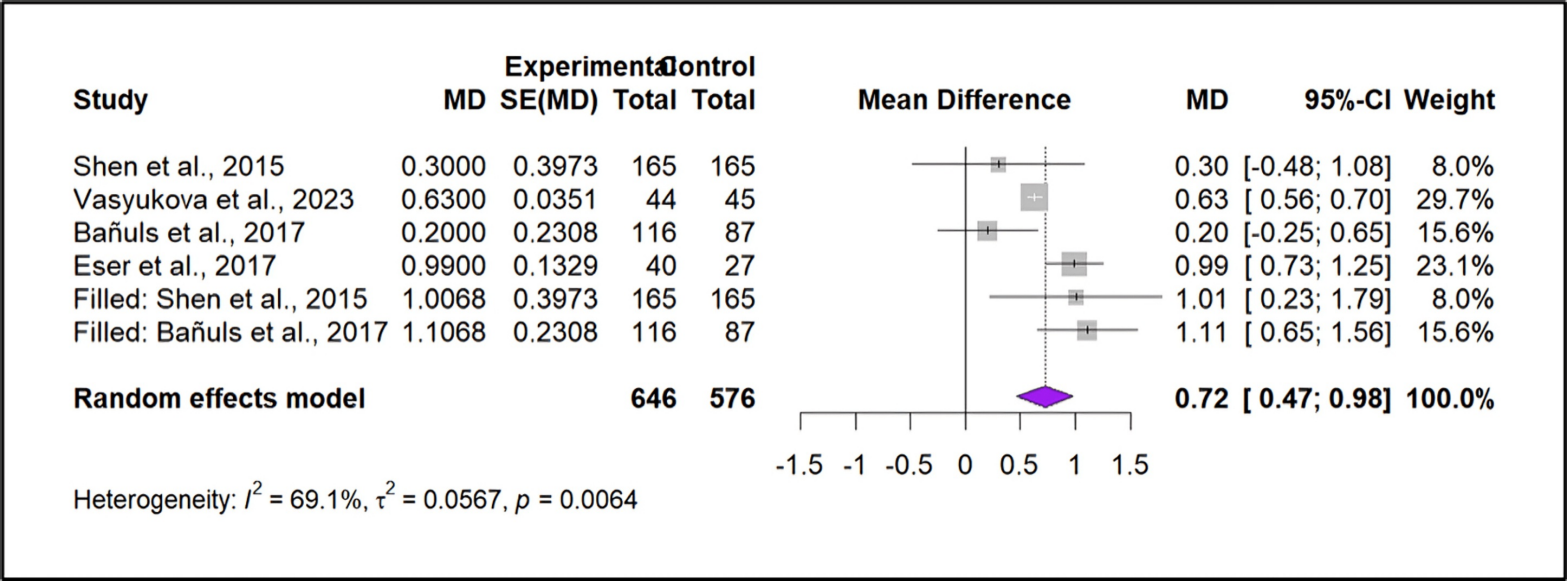
The second forest plot represents meta-analyses of the same data after Trim-and-Fill adjustment for publication bias Source: [25,27,31,36]

The funnel plot and Eggers test showed moderate to high heterogeneity (I² = 69.1%, p = 0.0064), but Tau² (0.0567) suggests that the differences in study estimates are not solely due to sampling error. However, the slight asymmetry observed in the funnel plot suggests the possibility of publication bias, where smaller studies with negative or neutral results may be missing. The Trim-and-Fill method (Figure 4) corrected for this bias by adding two studies, increasing the pooled effect size from MD = 0.62 to MD = 0.72. The adjusted effect size remained significant, indicating that publication bias is unlikely to have a major impact on the conclusions.

Discussion

All the studies included in the review examined various inflammatory markers in PCOS, revealing diverse levels of clinical significance. Inflammation plays a crucial role in the pathophysiology of PCOS, influencing both metabolic and reproductive functions. From 44 studies, we identified 94 inflammatory markers, which can be classified into 9 groups, such as cytokines & interleukins, acute phase proteins & inflammatory biomarkers, chemokines, growth factors, lipid mediators, oxidative stress & metabolic markers, apoptotic markers, metabolic & adipokines, and enzymes & proteins. Among these 94 markers, the most commonly used biomarkers, such as hs-CRP, IL-6, TNFα, CRP, Adiponectin, IL-18, VEGF, IL-8, IL-1β, SHBG, Leptin, and VCAM-1, are discussed in brief below.

Quantitative analysis of IL-6 in four case-control studies suggests a significant increase in IL-6 levels in PCOS patients with pooled MD 0.72 (p < 0.0001). Even though the meta-analysis reports the publication bias, we can observe that the impact of publication bias is not strong enough to change the overall significance of the results. The adjusted pooled mean difference (MD) using the random effects model is 0.724 (0.467; 0.981) after the Trim-and-Fill method added two studies to correct asymmetry, which indicates a significant increase in IL-6 levels in PCOS patients compared to controls. The heterogeneity (I² = 69.1%) suggests that study results vary substantially; therefore, the random effects model is appropriate due to heterogeneity. It is also important to note that heterogeneity is unavoidable in meta-analysis, as the primary goal of meta-analysis itself is to combine the results of various studies conducted in different contexts [8,10,51]. The sources of heterogenicity in this analysis are anticipated to be differences in BMI and age matching between cases and controls, differences in IL-6 measurement techniques, most importantly, genetic and ethnic differences since the data was from four different countries.

The most commonly investigated marker in the majority of the studies was hs-CRP, which is a systemic inflammation marker that was consistently elevated in PCOS patients across all reviewed studies [9,15,16]. Rudnicka et al. and Shen et al. demonstrated a positive correlation between hs-CRP, insulin resistance, dyslipidemia, and endothelial dysfunction [24,25]. While hs-CRP is commonly elevated in PCOS subjects, some variability exists based on patient characteristics such as Keskin et al. and Vasyukova et al. [26,27], who noted that obese PCOS patients exhibited significantly higher hs-CRP levels than their lean counterparts. The hs-CRP levels range from 2.4-6.8 mg/L in PCOS patients, whereas healthy individuals have levels between 0.5 and 2.0 mg/L; an increase of 2-4 times [9,16,24]. Compared to IL-6 and TNF-α, hs-CRP is a more stable, clinically accessible, and widely used marker of systemic inflammation. Unlike TNF-α, which primarily affects insulin signaling, hs-CRP is a broader marker for systemic inflammation and cardiovascular risk, which is consistently elevated across various studies [24,25].

Interleukin-6 (IL-6)

IL-6 is a pleiotropic cytokine involved in immune regulation, inflammation, and metabolism. It is primarily secreted by macrophages, adipocytes, and ovarian cells, and its dysregulation is strongly associated with metabolic and reproductive dysfunctions. In PCOS, IL-6 is linked to metabolic disturbances, including insulin resistance and obesity [8]. The study by Brenjian et al., Elbandrawy et al., Eser et al., and Rahimi et al. found PCOS subjects exhibited significantly higher IL-6 concentrations than controls, and Shen et al.'s study demonstrated that IL-6 was positively correlated with obesity and metabolic syndrome in PCOS. IL-6 levels were elevated in PCOS patients (2.5-5.8 pg/mL) as compared to controls (1.2-3.0 pg/mL), representing an approximately 1.8-fold increase [10,12,14,25,36]. Some studies observed that lean PCOS patients might exhibit only marginal IL-6 elevation compared to obese PCOS individuals. IL-6 is used for both diagnostic and prognostic applications in PCOS and is often considered superior due to its direct correlation with cardiovascular risk [8]. However, IL-6's role extends beyond systemic inflammation; it actively influences ovarian function, making it a more integrative biomarker for both metabolic and reproductive health in PCOS. When compared to TNF-α, IL-6 exhibits broader systemic effects, impacting both insulin resistance and ovarian physiology [10].

Tumor Necrosis Factor-Alpha (TNF-α)

TNF-α is a pro-inflammatory cytokine that plays a crucial role in immune response, inflammation, and metabolic regulation, which is secreted by macrophages and adipose tissue. Elevated levels of TNF-α contribute to a state of systemic inflammation, increasing both metabolic and reproductive dysfunctions. TNF-α is known to interfere with insulin signaling, which leads to insulin resistance, a core feature in PCOS. Additionally, it also results in ovarian dysfunction, where it disrupts steroidogenesis and follicular development. Several studies reported consistently elevated TNF-α levels in PCOS, such as studies by Sathyapalan et al. and Brenjian et al., which observed significantly higher TNF-α concentrations in women with PCOS, particularly in those with obesity and insulin resistance [8,10. Similarly, Jabarpour et al. and Elbandrawy et al. also confirmed increased TNF-α levels in PCOS cohorts, reinforcing its role as a pro-inflammatory cytokine in this condition [11,12]. TNF-α concentrations were 4.5-7.6 pg/mL in PCOS patients, compared to 2.0-4.5 pg/mL in controls, indicating a 1.5 to 2-fold elevation [11,12]. Studies also reported that TNF-α levels may vary based on BMI, metabolic phenotype, and severity of insulin resistance. Nasri et al. reported that TNF-α levels were markedly higher in women with PCOS and metabolic syndrome, suggesting a synergistic effect between obesity and inflammation [18]. Conversely, some lean PCOS patients exhibit only mild elevations in TNF-α, indicating that while TNF-α is a key player, its impact may be modulated by other metabolic factors [18]. When compared to IL-6, TNF-α appears to have a more direct effect on insulin resistance [11]. TNF-α is predominantly pro-inflammatory and acts primarily by impairing insulin receptor signaling. Additionally, compared to hs-CRP, TNF-α provides more mechanistic insights into the metabolic dysfunction seen in PCOS, whereas hs-CRP reflects generalized inflammation but does not directly influence insulin resistance and ovarian dysfunction as TNF-α does [10].

C-Reactive Protein (CRP)

CRP is an acute-phase protein primarily synthesized by the liver in response to inflammatory stimuli, particularly IL-6 and TNF-α. Elevated CRP levels in PCOS patients indicate the presence of chronic low-grade inflammation, which contributes to the syndrome’s metabolic, reproductive, and cardiovascular complications [12]. Studies report a strong association between increased CRP levels and key metabolic abnormalities in PCOS such as insulin resistance, obesity, dyslipidemia, and endothelial dysfunction. It has also been implicated in ovarian dysfunction in PCOS. Brenjian et al., Jabarpour et al., and Elbandrawy et al. also reported higher CRP concentrations [10-12]. Similarly, Pedroso et al. found that increased CRP levels correlated with worsening metabolic profiles, reinforcing its role as a marker of systemic inflammation [28]. CRP levels were 3.2-9.1 mg/L in PCOS, whereas controls had 0.8-3.5 mg/L, showing an approximately threefold increase in patient groups [12,28,43]. CRP levels in PCOS patients also show variability depending on BMI and metabolic phenotype. Shen et al. and Keskin et al. observed that obese PCOS patients had significantly higher CRP levels than their lean counterparts, suggesting that adipose tissue plays a major role in driving inflammation [25,26]. Compared to hs-CRP, CRP is a less sensitive marker of inflammation, and hs-CRP provides a more refined assessment of low-grade chronic inflammation [25,26]. When compared to other cytokines, such as IL-6 and TNF-α, CRP is a downstream marker rather than a direct inflammatory mediator. While IL-6 and TNF-α actively contribute to insulin resistance and ovarian dysfunction, CRP serves as an indicator of the overall inflammatory status [24].

Adiponectin

Adiponectin is an adipokine secreted primarily by adipose tissue, playing a crucial role in glucose metabolism, lipid regulation, and anti-inflammatory processes. Women with PCOS, particularly those with insulin resistance and obesity, often exhibit reduced levels of adiponectin compared to healthy controls [25]. Given that adiponectin enhances insulin sensitivity by promoting glucose uptake and fatty acid oxidation, its deficiency contributes to hyperinsulinemia, dyslipidemia, and metabolic dysfunction, all of which elevate PCOS symptoms. Studies indicate that lower adiponectin levels correlate with elevated androgens, menstrual irregularities, and anovulation. Unlike most inflammatory markers that are elevated in PCOS, adiponectin levels are significantly reduced [25,44]. Shen et al. reported lower adiponectin levels in PCOS patients, particularly those with metabolic syndrome [25]. Keskin et al. and Vasyukova et al. found an inverse correlation between adiponectin and BMI, insulin resistance, and C-reactive protein (CRP), indicating that obesity-driven inflammation further suppresses adiponectin production [26,27]. Adiponectin is significantly reduced in PCOS, with levels ranging from 5.8-12.1 μg/mL, compared to 9.0-16.5 μg/mL in controls, marking a 20-50% decrease [24-26]. Daan et al. showed that lean PCOS patients also exhibit lower adiponectin levels than non-PCOS controls, highlighting its role beyond obesity [38]. Compared to hs-CRP and TNF-α, which are elevated in PCOS and indicate a pro-inflammatory state, adiponectin functions as an anti-inflammatory regulator, where it counteracts insulin resistance and systemic inflammation effects by enhancing insulin sensitivity and reducing inflammation. When compared to IL-6, which is typically elevated in PCOS, adiponectin exhibits an inverse relationship, meaning that higher IL-6 levels are often accompanied by lower adiponectin concentrations. Additionally, VCAM-1 and ICAM-1, which are markers of endothelial dysfunction, correlate negatively with adiponectin levels, further linking its deficiency to cardiovascular risk in PCOS [13,22,44].

Interleukin-18 (IL-18)

IL-18 is a pro-inflammatory cytokine belonging to the IL-1 family, primarily secreted by macrophages and other immune cells, which is strongly implicated in metabolic disorders, including insulin resistance, obesity, and cardiovascular disease [10,11]. IL-18 has been identified as a major contributor to the chronic low-grade inflammation observed in PCOS, and the elevated levels correlated with increased oxidative stress, endothelial dysfunction, and hyperinsulinemia, all of which increase metabolic and reproductive abnormalities in PCOS patients. Beyond its metabolic effects, IL-18 also influences ovarian function and has been detected in ovarian follicles where increased IL-18 levels are associated with anovulation, menstrual irregularities, and hyperandrogenism [12,27,38]. Brenjian et al. and Jabarpour et al. reported markedly higher IL-18 concentrations in PCOS patients, with a strong correlation between IL-18, insulin resistance, and BMI [10,11]. Rahimi et al. demonstrated that IL-18 elevation was more pronounced in obese PCOS patients, reinforcing the role of adipose tissue-driven inflammation [14]. Ramamoorthy et al. and Eser et al. found that IL-18 levels were associated with menstrual cycle irregularities and hyperandrogenism, further implicating IL-18 in ovarian dysfunction [36,42]. IL-18 shows concentrations of 180-325 pg/mL in PCOS patients, whereas, in controls, it ranges from 90-190 pg/mL, indicating an approximately 1.8-fold increase [10,14,36]. Compared to IL-6 and TNF-α, which also contribute to systemic inflammation in PCOS, IL-18 appears to have a stronger association with insulin resistance. While IL-6 plays a dual role (both pro- and anti-inflammatory), IL-18 is predominantly pro-inflammatory and directly exacerbates metabolic and ovarian dysfunction. hs-CRP reflects systemic inflammation but does not directly influence insulin signaling or ovarian function, whereas IL-18 actively disrupts glucose metabolism, androgen secretion, and follicular maturation. Additionally, IL-18 shows a positive correlation with TNF-α, IL-6, and oxidative stress markers. Compared to adiponectin, which is decreased in PCOS. IL-18 functions in the opposite direction, further emphasizing the inflammatory imbalance seen in the syndrome [11,27,38].

Vascular Endothelial Growth Factor (VEGF)

VEGF is a potent angiogenic cytokine that plays a crucial role in blood vessel formation, endothelial cell proliferation, and vascular permeability. It is primarily secreted by granulosa cells, the ovarian stroma, and adipose tissue. VEGF is essential for normal ovarian function, regulating follicular development, corpus luteum formation, and ovarian angiogenesis, and its dysregulation has been implicated in PCOS, particularly in relation to ovarian hyperstimulation, inflammation, and metabolic dysfunction [8,38]. Sathyapalan et al. reported that PCOS patients had higher circulating VEGF levels, with a strong correlation between VEGF, insulin resistance, and BMI [8]. Heidar et al. and Hatziagelaki et al. demonstrated that VEGF expression was upregulated in both the serum and ovarian follicular fluid of PCOS patients, indicating its direct role in abnormal folliculogenesis [17,41]. Daan et al. and Kałużna et al. observed that VEGF levels were significantly higher in women with PCOS and hyperandrogenism, suggesting a relationship between VEGF and excessive androgen production [38,39]. Similarly, Elbandrawy et al. found that increased VEGF levels were associated with a higher risk of endothelial dysfunction and cardiovascular disease in PCOS patients [12]. VEGF levels were 250-570 pg/mL in PCOS patients compared to 120-310 pg/mL in controls, representing a 1.8 to 2-fold increase [8,17,41]. Compared to IL-6 and TNF-α, which primarily mediate systemic inflammation, VEGF is more directly involved in ovarian dysfunction and vascular abnormalities in PCOS. While IL-6 and TNF-α promote insulin resistance and chronic inflammation, VEGF plays a dual role by contributing to both inflammation and pathological ovarian angiogenesis [7,8]. Compared to hs-CRP, VEGF provides more mechanistic insights into ovarian dysfunction rather than serving as a broad marker of systemic inflammation. hs-CRP reflects generalized inflammation, whereas VEGF directly contributes to follicular arrest and abnormal blood vessel formation in PCOS. VEGF also exhibits a positive correlation with oxidative stress markers, reinforcing its role in vascular complications and endothelial dysfunction. Compared to adiponectin, which has anti-inflammatory and insulin-sensitizing properties, VEGF acts in the opposite direction, promoting inflammation, hyperpermeability, and endothelial dysfunction [9,17].

Interleukin-8 (IL-8)

IL-8, also known as CXCL8, is a pro-inflammatory chemokine that plays a significant role in immune cell recruitment, angiogenesis, and tissue remodeling. It is primarily secreted by monocytes, macrophages, endothelial cells, and granulosa cells, acting as a potent mediator of inflammation and oxidative stress [8,17,52]. IL-8 contributes to the chronic low-grade inflammation that underlies metabolic dysfunction, ovarian abnormalities, insulin resistance, and endothelial inflammation, all of which are key pathophysiological features of PCOS. IL-8, if found in both serum and follicular fluid, suggests a direct role in follicular growth arrest and impaired steroidogenesis [8,42]. Multiple studies, such as Sathyapalan et al. and Heidar et al., reported higher IL-8 concentrations in PCOS patients, particularly in those with obesity and insulin resistance [8,17]. Brenjian et al. found that IL-8 levels were significantly elevated in follicular fluid, correlating with poor oocyte quality and anovulation [10]. Elbandrawy et al. and Jamilian et al. observed a strong association between IL-8 elevation and increased oxidative stress markers, further reinforcing its role in PCOS-related chronic inflammation [7,12]. Daan et al. demonstrated that IL-8 levels were higher in women with PCOS and hyperandrogenism, suggesting a link between ovarian inflammation and androgen excess [38]. IL-8 concentrations range from 6.3-18.4 pg/mL in PCOS, whereas in controls, they are 3.2-9.1 pg/mL, showing a twofold elevation. Compared to IL-6 and TNF-α, which primarily mediate systemic inflammation, IL-8 appears to have a more localized impact on ovarian dysfunction and angiogenesis in PCOS. While IL-6 contributes to insulin resistance and chronic inflammation. IL-8 is directly involved in follicular atresia, ovarian stromal remodeling, and abnormal blood vessel formation [17]. When compared to other markers of systemic inflammation, IL-8 provides a more specific insight into ovarian and vascular inflammation in PCOS. Additionally, IL-8 correlates positively with TNF-α and VEGF, indicating a synergistic role in promoting endothelial dysfunction and oxidative stress. Unlike adiponectin, which is decreased in PCOS and has anti-inflammatory effects, IL-8 follows the opposite trend, with higher levels contributing to worsening metabolic and ovarian dysfunction [10,11,27].

Interleukin-1 Beta (IL-1β)

IL-1β is a pro-inflammatory cytokine belonging to the IL-1 family, primarily secreted by monocytes, macrophages, and ovarian cells in response to inflammatory stimuli. It plays a crucial role in initiating and amplifying inflammatory responses, oxidative stress, and immune signaling. It is a key mediator in chronic low-grade inflammation in PCOS, where it contributes to metabolic dysfunction, insulin resistance, and ovarian abnormalities. It is also involved in follicular development and ovulation, but its overexpression in PCOS disrupts steroidogenesis and oocyte maturation, leading to anovulation and menstrual irregularities. IL-1β promotes androgen production in ovarian theca cells and interferes with insulin signaling pathways [10,27]. IL-1β levels are significantly elevated in women with PCOS compared to healthy controls, as confirmed by studies of Brenjian et al. and Eser et al., who found higher IL-1β levels in PCOS patients, correlating with hyperandrogenism and insulin resistance [10,36]. Fereidouni et al. demonstrated that IL-1β was significantly upregulated in the serum and follicular fluid of PCOS patients undergoing fertility treatment, affecting oocyte quality and ovulatory response [13]. Rahimi et al. [14] and Daan et al. [38] observed a strong correlation between IL-1β and oxidative stress markers, whereas Shen et al. [25] found that obese PCOS patients exhibited markedly higher IL-1β levels, while lean PCOS patients showed moderate elevations, suggesting a synergistic relationship between obesity and IL-1β-driven inflammation [14,25,38]. IL-1β levels are 3.5-7.2 pg/mL in PCOS patients compared to 1.5-3.8 pg/mL in controls, reflecting a 1.5 to 2-fold increase [10,13,14]. Compared to IL-6 and TNF-α, IL-1β has a more localised impact on ovarian dysfunction and hyperandrogenism. While IL-6 plays a dual role (both pro- and anti-inflammatory), IL-1β is strictly pro-inflammatory, leading to ovarian inflammation and androgen excess. When compared to hs-CRP, IL-1β provides deeper mechanistic insights into PCOS pathophysiology, particularly in ovarian and metabolic dysfunction. Additionally, IL-1β strongly correlates with TNF-α, IL-18, and oxidative stress markers, indicating that it amplifies the inflammatory cascade in PCOS [8,14].

Sex Hormone-Binding Globulin (SHBG)

SHBG is a glycoprotein primarily synthesized in the liver that plays a key role in regulating sex hormone bioavailability. In PCOS, SHBG levels are often reduced, leading to an increase in free testosterone, which exacerbates hyperandrogenic symptoms such as hirsutism, acne, and menstrual irregularities. SHBG is a critical marker of metabolic and hormonal balance, with its levels being influenced by insulin, inflammatory cytokines, and liver function. Reduced SHBG contributes to ovarian dysfunction, insulin resistance, and cardiovascular risk in PCOS [18,24,25]. Unlike pro-inflammatory markers, such as IL-6, TNF-α, and IL-1β, which are elevated in PCOS, SHBG levels are significantly reduced. Rudnicka et al. and Shen et al. found that SHBG levels were markedly lower in women with PCOS, correlating with hyperandrogenism and insulin resistance [24,25]. Nasri et al. demonstrated an inverse relationship between SHBG and hs-CRP, further linking SHBG suppression to chronic inflammation [18]. Keskin et al. observed that obese PCOS patients had the lowest SHBG levels, supporting the negative impact of insulin resistance on SHBG production [26]. Kałużna et al. [31] and Bañuls et al. [39] reported that SHBG suppression was more severe in women with PCOS and metabolic syndrome, highlighting its role in PCOS-related cardiometabolic risk [31,39]. SHBG is notably reduced in PCOS, with levels ranging from 10-45 nmol/L, compared to 30-90 nmol/L in healthy individuals, showing a 40-60% decrease [18, 24, 26]. Compared to IL-6 and TNF-α, which indicate chronic inflammation, SHBG is a negative marker that is it decreases, worsening insulin resistance and hyperandrogenism. While IL-6 and TNF-α actively drive inflammation, SHBG reduction is a consequence of metabolic and hormonal imbalances in PCOS. When compared to hs-CRP, which serves as a broad marker of systemic inflammation, SHBG provides more specific insights into androgen excess and insulin resistance [26,39]. Additionally, low SHBG correlates strongly with hyperandrogenic symptoms, making it an essential clinical marker for diagnosing and managing PCOS-related hyperandrogenism. Unlike adiponectin, which is also decreased in PCOS but has anti-inflammatory properties, SHBG primarily regulates hormonal balance rather than inflammation. However, its reduction worsens metabolic and cardiovascular complications, reinforcing the bidirectional relationship between metabolic dysfunction and inflammation [22,24,25].

Leptin

Leptin is an adipokine primarily secreted by adipose tissue that plays a crucial role in appetite regulation, energy metabolism, and reproductive function. It acts on the hypothalamus to regulate food intake and energy expenditure while also influencing glucose metabolism, insulin sensitivity, and ovarian function. Leptin is involved in both metabolic regulation and chronic inflammation, making it a crucial marker in PCOS. Leptin levels are significantly elevated in women with PCOS, particularly in those with obesity and metabolic syndrome [5,38]. Studies like Shen et al. and Daan et al. found that leptin levels were significantly higher in PCOS patients, correlating with BMI, insulin resistance, and other inflammatory markers [25,38]. Rudnicka et al. demonstrated that leptin was inversely correlated with SHBG, indicating a strong link between leptin-induced hyperinsulinemia and androgen excess, and Kałużna et al. observed that women with PCOS and central obesity exhibited the highest leptin levels, reinforcing the role of visceral adiposity in leptin dysregulation [24,39]. Similarly, Goswami et al. found that leptin was positively associated with hs-CRP and TNF-α, suggesting that leptin-driven inflammation contributes to metabolic and cardiovascular complications in PCOS [37]. Leptin is elevated in PCOS, with levels ranging from 15-38 ng/mL, whereas controls exhibit 5-20 ng/mL, marking a 2 to 3-fold increase, particularly in obese PCOS patients [25,38,37]. Compared to IL-6 and TNF-α, leptin is both a metabolic and inflammatory regulator. While IL-6 and TNF-α contribute directly to insulin resistance and inflammation, leptin plays an additional role in reproductive dysfunction by influencing ovarian steroidogenesis and follicular maturation [7,50]. When compared to adiponectin, leptin acts in the opposite direction, with higher levels promoting insulin resistance and metabolic dysfunction. The leptin-to-adiponectin ratio has been proposed as a better predictor of metabolic complications in PCOS than either marker alone. Unlike hs-CRP, leptin provides more specific insights into obesity driven metabolic dysfunction, making it a valuable predictor of insulin resistance and cardiovascular risk in PCOS patients [25,37,38].

Vascular Cell Adhesion Molecule-1 (VCAM-1)

VCAM-1 is an endothelial adhesion molecule that plays a crucial role in vascular inflammation, immune cell recruitment, and endothelial dysfunction. As already discussed above, in PCOS, chronic low-grade inflammation and metabolic disturbances contribute to endothelial dysfunction, increasing the risk of CVD [35, 31]. This heightened cardiovascular risk in PCOS is primarily driven by insulin resistance, chronic inflammation, and endothelial dysfunction. VCAM-1 serves as a key mediator in these processes, as it reflects vascular inflammation and the progression of atherosclerotic changes in PCOS patients [31]. Several studies have demonstrated a significant increase in VCAM-1 levels in PCOS patients compared to healthy controls. Reported levels range from 420-800 ng/mL in PCOS individuals, whereas controls exhibit levels between 250-500 ng/mL, indicating a 1.6-2 fold elevation [31,38,50]. Kałużna et al. and Bañuls et al. specifically noted that VCAM-1 levels were markedly higher in women with PCOS, particularly those with metabolic syndrome, suggesting a strong link between vascular inflammation and insulin resistance [31,39]. Additionally, Daan et al. reported a significant correlation between VCAM-1 and pro-inflammatory markers, such as IL-6, TNF-α, and oxidative stress indicators, reinforcing its role as a marker of endothelial dysfunction [38]. Goswami et al. found that obese PCOS patients exhibited the highest VCAM-1 levels, suggesting that adiposity exacerbates endothelial dysfunction [37]. Koleva et al. found that VCAM-1 levels were inversely correlated with adiponectin, which signifies the link between metabolic dysfunction and vascular inflammation in PCOS [50]. Compared to IL-6 and TNF-α, which primarily indicate systemic inflammation, VCAM-1 is a more specific marker of endothelial dysfunction. While IL-6 and TNF-α contribute to metabolic abnormalities and chronic inflammation [31,37]. VCAM-1 directly participates in vascular inflammation and atherosclerosis progression. Additionally, compared to hs-CRP, a broad marker of systemic inflammation, VCAM-1 provides a more precise measure of vascular injury, making it a strong predictor of cardiovascular complications in PCOS. Unlike adiponectin, which has anti-inflammatory and insulin-sensitizing properties and is reduced in PCOS, VCAM-1 is elevated, reflecting an increased risk of vascular inflammation and cardiovascular events.

To condense the extensive qualitative nature of this review, we have focused on briefly discussing only the most significant markers identified through the systematic review while providing a more detailed analysis of those markers that were frequently reported across the majority of studies. These findings are consistent with those of a previously published systematic review on inflammatory markers in PCOS, which reported elevated levels of circulating inflammatory markers in affected individuals. Notably, that review was conducted in 2010, prior to the introduction of the PRISMA guidelines. In contrast, our current systematic review offers a comprehensive synthesis of recent evidence from the past decade and will serve as a valuable resource for ongoing and future research in PCOS and women’s health [53].

Although we adhered to rigorous scientific standards and followed PRISMA guidelines in conducting this systematic review and meta-analysis, certain limitations in the current approach need to be acknowledged. We have only included studies published in the last 10 years so that future studies are recommended to compile evidence from the inception of research to the present. Furthermore, for observational studies, adherence to MOOSE guidelines for reporting should also be considered in future research [54]. The findings of this systematic review and meta-analysis will provide valuable guidance for ongoing and future research efforts. The overall summary of the compiled evidence indicates that measuring inflammatory markers in PCOS is a promising approach that can help reduce PCOS-associated complications and improve health outcomes and treatment effectiveness.

## Conclusions

Chronic low-grade inflammation is a consistent symptom of PCOS. The results of this review suggest that IL-6 may serve as a valuable diagnostic and prognostic marker for assessing inflammation in PCOS. Additionally, other key inflammatory biomarkers, including high-sensitivity C-reactive protein (hs-CRP), tumor necrosis factor-alpha (TNF-α), C-reactive protein (CRP), adiponectin, interleukin-18 (IL-18), vascular endothelial growth factor (VEGF), interleukin-8 (IL-8), interleukin-1 beta (IL-1β), sex hormone-binding globulin (SHBG), leptin, and vascular cell adhesion molecule-1 (VCAM-1), can also provide insight into the inflammatory status and overall metabolic health of PCOS patients.

## Appendices

**Table 6:** Quality assessment using the ROBINS-1 tool for non-randomized trials [22-23]

Study 1	Risk of Bias Domain	Assessment Criteria	Risk Level (Low/Moderate/High)	Comments/Justification
Szczuko et al., 2017 [23]	Bias due to confounding	Were baseline characteristics and potential confounders measured and adjusted for?	Moderate	Low risk of bias
	Bias in selection of participants	Was participant selection biased, or was it well-defined (e.g., inclusion/exclusion criteria, dropouts)?	Moderate	
	Bias in classification of interventions			
	Bias due to deviations from intended interventions	Were participants compliant? Were there deviations?	Low	
	Bias due to missing data	Were dropouts/exclusions reported? Were they balanced across groups?	Moderate	
	Bias in measurement of outcomes	Were outcomes assessed objectively and using validated methods?	Low	
	Bias in selection of reported results	Were all planned analyses and outcomes reported?	Low	
Study 2	Risk of Bias Domain	Assessment Criteria	Risk Level (Low/Moderate/High)	Comments/Justification
Covington et al., 2016 [22]	Bias due to confounding	Were baseline characteristics and potential confounders measured and adjusted for?	Moderate	Low risk of bias
	Bias in selection of participants	Was participant selection biased, or was it well-defined (e.g., inclusion/exclusion criteria, dropouts)?	Low	
	Bias in classification of interventions		Moderate	
	Bias due to deviations from intended interventions	Were participants compliant? Were there deviations?	Low	
	Bias due to missing data	Were dropouts/exclusions reported? Were they balanced across groups?	Low	
	Bias in measurement of outcomes	Were outcomes assessed objectively and using validated methods?	Low	
	Bias in selection of reported results	Were all planned analyses and outcomes reported?	Low	

**Table 7:** Quality assessment using the Cochrane risk-of-bias (RoB-2) tool for randomized controlled trials [7-21]

Study 1	Domain	Question	Response options (Y = yes, Py = Probably yes, PN = Probably No, N = No)	Risk-of-bias judgment
Jamilian et al., 2018 [7]	Randomization Process	Was the allocation sequence random?	Y	Low
	Randomization Process	Was the allocation sequence concealed until participants were enrolled and assigned to interventions?	Y	
	Baseline Differences	Did baseline differences between intervention groups suggest a problem with the randomization process?	PY	
	Blinding	Were participants, personnel, and outcome assessors blinded?	Y	
	Incomplete Outcome Data	Were dropout rates and reasons reported?	PN	
	Selective Reporting	Were all prespecified outcomes reported?	PY	
Study 2	Domain	Question	Response options	Risk-of-bias judgment
Sathyapalan et al., 2017 [8]	Randomization Process	Was the allocation sequence random?	Yes	Low
	Randomization Process	Was the allocation sequence concealed until participants were enrolled and assigned to interventions?	No	
	Baseline Differences	Did baseline differences between intervention groups suggest a problem with the randomization process?	No	
	Blinding	Were participants, personnel, and outcome assessors blinded?	Yes	
	Incomplete Outcome Data	Were dropout rates and reasons reported?	Yes	
	Selective Reporting	Were all prespecified outcomes reported?	Yes	
Study 3	Domain	Question	Response options	Risk-of-bias judgment
Abbasi et al., 2023 [9]	Randomization Process	Was the allocation sequence random?	Yes	Low
	Randomization Process	Was the allocation sequence concealed until participants were enrolled and assigned to interventions?	Yes	
	Baseline Differences	Did baseline differences between intervention groups suggest a problem with the randomization process?	No	
	Blinding	Were participants, personnel, and outcome assessors blinded?	Yes	
	Incomplete Outcome Data	Were dropout rates and reasons reported?	Yes	
	Selective Reporting	Were all prespecified outcomes reported?	No	
Study 4	Domain	Question	Response options	Risk-of-bias judgment
Brenjian et al., 2020 [10]	Randomization Process	Was the allocation sequence random?	Yes	Low
	Randomization Process	Was the allocation sequence concealed until participants were enrolled and assigned to interventions?	Yes	
	Baseline Differences	Did baseline differences between intervention groups suggest a problem with the randomization process?	No	
	Blinding	Were participants, personnel, and outcome assessors blinded?	Yes	
	Incomplete Outcome Data	Were dropout rates and reasons reported?	Yes	
	Selective Reporting	Were all prespecified outcomes reported?	PY	
Study 5	Domain	Question	Response options	Risk-of-bias judgment
Jabarpour et al., [11]	Randomization Process	Was the allocation sequence random?	Yes	Low
	Randomization Process	Was the allocation sequence concealed until participants were enrolled and assigned to interventions?	Yes	
	Baseline Differences	Did baseline differences between intervention groups suggest a problem with the randomization process?	PN	
	Blinding	Were participants, personnel, and outcome assessors blinded?	Yes	
	Incomplete Outcome Data	Were dropout rates and reasons reported?	Yes	
	Selective Reporting	Were all prespecified outcomes reported?	PY	
Study 6	Domain	Question	Response options	Risk-of-bias judgment
Elbandrawy et al., 2022 [12]	Randomization Process	Was the allocation sequence random?	Y	Low
	Randomization Process	Was the allocation sequence concealed until participants were enrolled and assigned to interventions?	PY	
	Baseline Differences	Did baseline differences between intervention groups suggest a problem with the randomization process?	No	
	Blinding	Were participants, personnel, and outcome assessors blinded?	PY	
	Incomplete Outcome Data	Were dropout rates and reasons reported?	No	
	Selective Reporting	Were all prespecified outcomes reported?	Yes	
Study 7	Domain	Question	Response options	Risk-of-bias judgement
Fereidouni et al., 2024 [13]	Randomization Process	Was the allocation sequence random?	PY	Low
	Randomization Process	Was the allocation sequence concealed until participants were enrolled and assigned to interventions?	Y	
	Baseline Differences	Did baseline differences between intervention groups suggest a problem with the randomization process?	N	
	Blinding	Were participants, personnel, and outcome assessors blinded?	Y	
	Incomplete Outcome Data	Were dropout rates and reasons reported?	Y	
	Selective Reporting	Were all prespecified outcomes reported?	Y	
Study 8	Domain	Question	Response options	Risk-of-bias judgment
Rahimi et al., 2022 [14]	Randomization Process	Was the allocation sequence random?	N	Some concerns
	Randomization Process	Was the allocation sequence concealed until participants were enrolled and assigned to interventions?	PN	
	Baseline Differences	Did baseline differences between intervention groups suggest a problem with the randomization process?	Y	
	Blinding	Were participants, personnel, and outcome assessors blinded?	PN	
	Incomplete Outcome Data	Were dropout rates and reasons reported?	Y	
	Selective Reporting	Were all prespecified outcomes reported?	Y	
Study 9	Domain	Question	Response options	Risk-of-bias judgement
Verma et al., 2024 [15]	Randomization Process	Was the allocation sequence random?	Y	Some concerns/Low/High
	Randomization Process	Was the allocation sequence concealed until participants were enrolled and assigned to interventions?	PY	
	Baseline Differences	Did baseline differences between intervention groups suggest a problem with the randomization process?	PN	
	Blinding	Were participants, personnel, and outcome assessors blinded?	PY	
	Incomplete Outcome Data	Were dropout rates and reasons reported?	No	
	Selective Reporting	Were all prespecified outcomes reported?	Y	
Study 10	Domain	Question	Response options	Risk-of-bias judgment
Asemi et al., 2015 [16]	Randomization Process	Was the allocation sequence random?	Y	Low
	Randomization Process	Was the allocation sequence concealed until participants were enrolled and assigned to interventions?	PY	
	Baseline Differences	Did baseline differences between intervention groups suggest a problem with the randomization process?	N	
	Blinding	Were participants, personnel, and outcome assessors blinded?	Y	
	Incomplete Outcome Data	Were dropout rates and reasons reported?	Y	
	Selective Reporting	Were all prespecified outcomes reported?	Y	
Study 11	Domain	Question	Response options	Risk-of-bias judgment
Heidar et al., 2020 [17]	Randomization Process	Was the allocation sequence random?	Y	Low
	Randomization Process	Was the allocation sequence concealed until participants were enrolled and assigned to interventions?	Y	
	Baseline Differences	Did baseline differences between intervention groups suggest a problem with the randomization process?	PN	
	Blinding	Were participants, personnel, and outcome assessors blinded?	Y	
	Incomplete Outcome Data	Were dropout rates and reasons reported?	Y	
	Selective Reporting	Were all prespecified outcomes reported?	Y	
Study 12	Domain	Question	Response options	Risk-of-bias judgment
Nasri et al., 2018 [18]	Randomization Process	Was the allocation sequence random?	Y	Low
	Randomization Process	Was the allocation sequence concealed until participants were enrolled and assigned to interventions?	PY	
	Baseline Differences	Did baseline differences between intervention groups suggest a problem with the randomization process?	Y	
	Blinding	Were participants, personnel, and outcome assessors blinded?	Y	
	Incomplete Outcome Data	Were dropout rates and reasons reported?	PY	
	Selective Reporting	Were all prespecified outcomes reported?	Y	
Study 13	Domain	Question	Response options	Risk-of-bias judgment
Esmaeilinezhad et al., 2020 [19]	Randomization Process	Was the allocation sequence random?	Yes	Low
	Randomization Process	Was the allocation sequence concealed until participants were enrolled and assigned to interventions?	Yes	
	Baseline Differences	Did baseline differences between intervention groups suggest a problem with the randomization process?	NO	
	Blinding	Were participants, personnel, and outcome assessors blinded?	Yes	
	Incomplete Outcome Data	Were dropout rates and reasons reported?	Yes	
	Selective Reporting	Were all prespecified outcomes reported?	Yes	
Study 14	Domain	Question	Response options	Risk-of-bias judgment
Zangeneh et al., 2017 [20]	Randomization Process	Was the allocation sequence random?	Yes	Low
	Randomization Process	Was the allocation sequence concealed until participants were enrolled and assigned to interventions?	No	
	Baseline Differences	Did baseline differences between intervention groups suggest a problem with the randomization process?	NO	
	Blinding	Were participants, personnel, and outcome assessors blinded?	Yes	
	Incomplete Outcome Data	Were dropout rates and reasons reported?	Yes	
	Selective Reporting	Were all prespecified outcomes reported?	Yes	
Study 15	Domain	Question	Response options	Risk-of-bias judgment
Alissa et al., 2021 [21]	Randomization Process	Was the allocation sequence random?	PN	Some concerns
	Randomization Process	Was the allocation sequence concealed until participants were enrolled and assigned to interventions?	N	
	Baseline Differences	Did baseline differences between intervention groups suggest a problem with the randomization process?	N	
	Blinding	Were participants, personnel, and outcome assessors blinded?	N	
	Incomplete Outcome Data	Were dropout rates and reasons reported?	Y	
	Selective Reporting	Were all prespecified outcomes reported?	Y	

**Table 8:** Quality assessment using the New-Castle Ottawa scale for case-control studies [24-36]

Study Title	Rate the selection process of study participants on a scale of 0 to 4 stars. Consider factors like representativeness of the sample and ascertainment of exposure	Evaluate the comparability of study groups on a scale of 0 to 2 stars. Focus on the adjustment for confounding variables.	Assess the outcome or exposure measurement on a scale of 0 to 3 stars. Consider follow-up duration and completeness of data.	Sum up the stars from the three domains to calculate the total score (max = 9 stars).	Based on the total stars, assign a quality rating: Poor (0-3), Fair (4-6), Good (7-8), Very Good (9)
Shen et al., 2015 [25]	3	2	2	7	Good
Keskin et al., 2014 [26]	3	2	3	8	Good
Vasyukova et al., 2023 [27]	3	2	2	7	Good
Pedroso et al., 2015 [28]	4	2	3	9	Very Good
Demi̇r et al., 2019 [29]	3	2	2	7	Good
Wang et al., 2017 [30]	3	2	3	8	Good
Ganie et al., 2016 [35]	2	2	3	6	Fair
Bañuls et al., 2017 [31]	4	2	3	9	Very Good
Apaydın et al., 2021 [32]	3	2	2	7	Good
Niu et al., 2017 [33]	3	2	3	8	Good
Akan et al., 2022 [34]	3	2	3	8	Good
Ganie et al., 2016 [35]	3	2	2	7	Good
Eser et al., 2017 [36]	3	1	3	6	Fair
Rudnicka et al., 2020 [24]	3	2	2	7	Good

**Table 9:** Quality assessment using the New-Castle Ottawa scale for cross-sectional studies [37-50]

Study title	Rate the representativeness of the sample, sample size, and response rate. A maximum of 5 stars can be awarded.	Evaluate whether the study accounted for confounding factors. Provide up to 2 stars based on the methods used to adjust for these variables.	Assess the validity and reliability of the outcome measurement. Include considerations like consistency and follow-up where applicable. Assign up to 3 stars.	Add the scores from all three domains. The maximum score is 10 stars.	Assign a rating based on the total stars: Poor (0-3), Fair (4-6), Good (7-8), Very Good (9-10).
Goswami et al., 2021 [37]	4	1	2	7	Good
Daan et al., 2016 [38]	3	2	2	7	Good
Kałużna et al., 2020 [39]	4	2	2	8	Good
Tola et al., 2017 [40]	3	2	2	7	Good
Hatziagelaki et al., 2020 [41]	3	1	3	7	Good
Ramamoorthy et al., 2019 [42]	4	2	3	9	Very Good
Shorakae et al., 2018 [44]	3	2	2	7	Good
Atnil et al., 2022 [45]	3	1	2	6	Fair
Asfuroğlu et al., 2021 [46]	4	2	2	8	Good
Bicer et al., 2017 [47]	3	2	3	9	Very Good
Amjadi et al., 2015 [48]	3	1	3	7	Good
Temur et al., 2017 [49]	4	2	3	9	Very Good
Koleva et al., 2016 [50]	3	1	2	6	Fair
